# T-helper cell polarisation following severe polytrauma

**DOI:** 10.1186/2197-425X-3-S1-A848

**Published:** 2015-10-01

**Authors:** HDT Torrance, K Brohi, G Warnes, HC Owen, CJ Hinds, DJ Pennington, MJ O'Dwyer

**Affiliations:** Barts & the London School of Medicine, QMUL, William Harvey Research Institute, London, United Kingdom; Barts & the London School of Medicine, QMUL, Blizard Institute, London, United Kingdom; Barts Health NHS Trust, Trauma & Critical Care, London, United Kingdom

## Introduction

Severe polytrauma induces an immunosuppressive response and is associated with a very high incidence of nosocomial infections. Previous studies have inferred that this detrimental immune response results from polarisation of the T helper (T_h_) response towards an anti-inflammatory, T_H_2 dominated, response at the expense of a bactericidal, T_h_1 response [1].

## Objectives

1) To define alterations in T_H_ cell subsets following severe blunt polytrauma.

## Methods

Patients presenting to the emergency department within 2 hours of severe polytrauma were eligible if intubated either at the scene or in ED. Isolated head injuries and those not expected to survive 24 hours were excluded. EDTA anti-coagulated blood was drawn at 0hr (within 2 hours of injury), at 24 and 72hrs. Samples were immediately lysed, washed, stained and analysed using a standardised human 8-colour T_H_ 1, 2 & 17 panel [2] on an LSR II flow cytometer. A paired white cell count differential was obtained at each sampling point. Patients were followed until discharge or death. Data were analysed using non-parametric statistics, with results presented as median and IQR.

## Results

**Figure 1:**
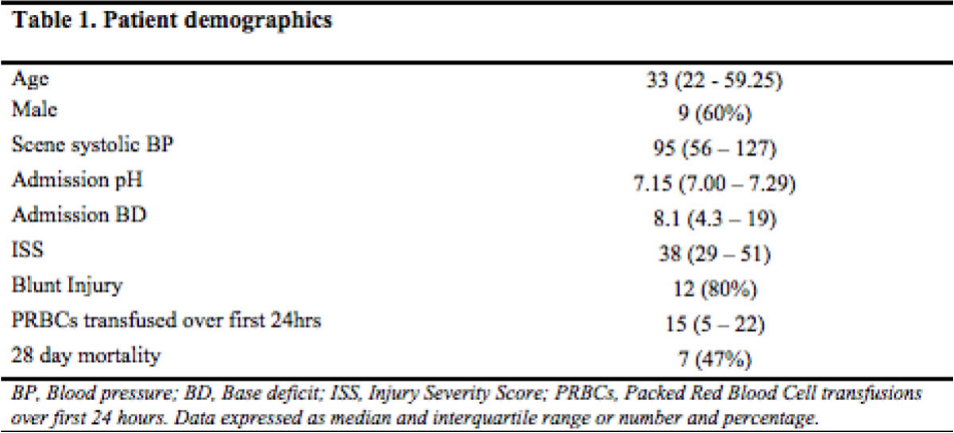
Figure 1

**Figure 2:**
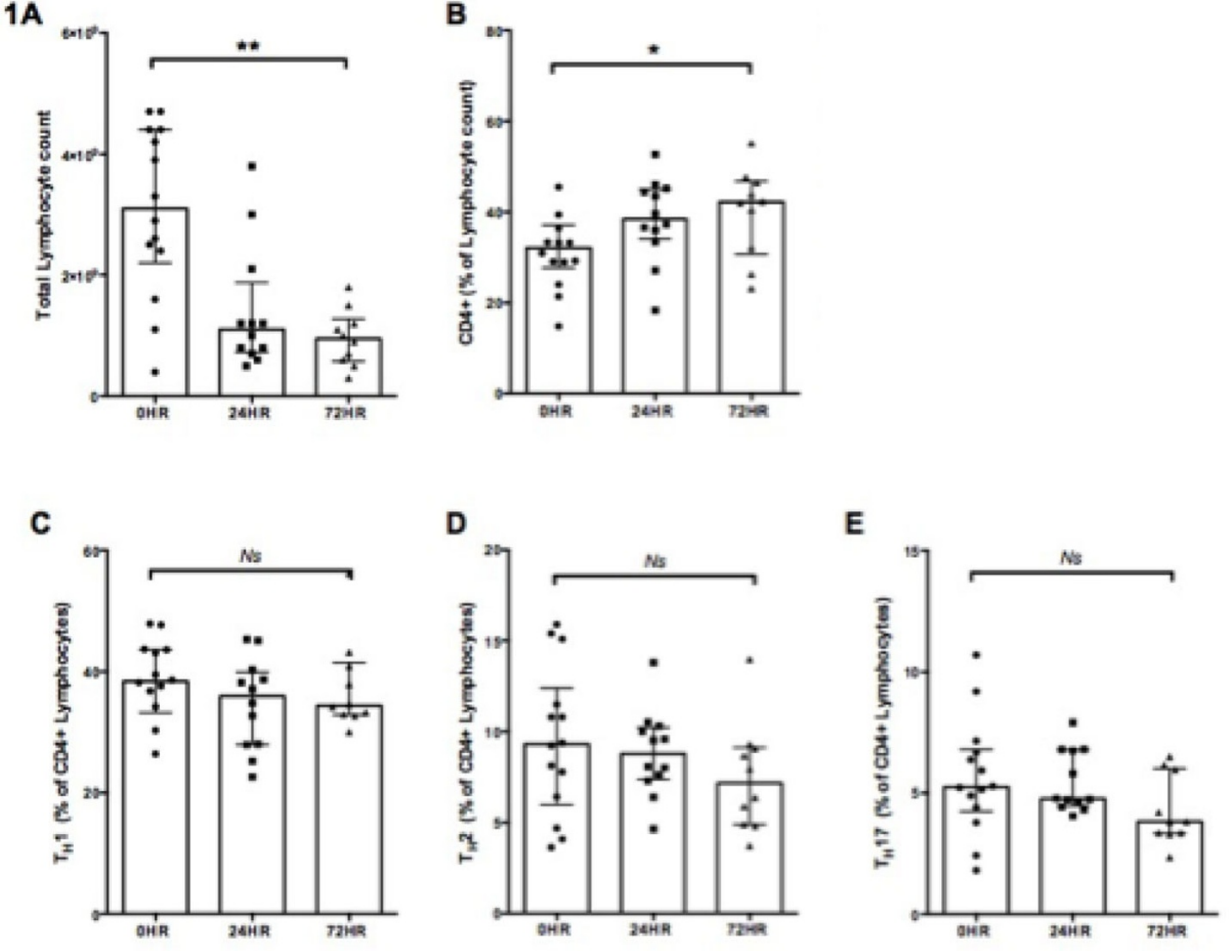
Figure 2

15 consecutive severe polytrauma patients requiring Intensive Care Unit (ICU) admission were recruited. Demographic and clinical data are outlined in Figure 1. Twelve (80%) lymphocytosis (3.3x10^9^/L, 2.5 - 4.4x10^9^/L) (Figyre 2A). At 72 hours leukocytes had fallen (*P* < 0.01, figure 2A) such that 6 (54%) of those surviving were lymphopenic (0.9x10^9^/L, 0.6 - 1.2x10^9^/L). Circulating CD4^+^ (*P* = 0.01; Figure 2B) and CD4^+^CD25^+^ (*P* < 0.05) lymphocytes increased over 72 hours. When expressed as a percentage of total circulating lymphocytes no significant change in the proportions of the T_H_ 1, 2 & 17 subpopulations was detected (Figure 2C-E).

## Conclusions

Severe polytrauma patients swiftly become lymphopenic. Although a failure to normalise this during the ICU stay correlates with higher mortality [3] our study of T_H_ cell subtypes demonstrates no evidence of a switch to a detrimental anti-inflammatory T_H_2 subtype at the expense of the potentially protective bactericidal T_H_1 subtype.

## Grant Acknowledgment

Royal College of Surgeons of England, Barts & the London Charity.
